# 1^st^Symposium on Y-Chromosome Human Proteome Project

**DOI:** 10.22074/cellj.2015.502

**Published:** 2015-01-13

**Authors:** Muhammad Irfan-Maqsood

**Affiliations:** 1 Department of Stem Cells Research and Regenerative Medicine, ACECR Mashhad Branch, Mashhad, Iran; 2 Department of Biology, Faculty of Sciences, Ferdowsi University of Mashhad, Mashhad, Iran; 3 Nastaran Centre for Cancer Prevention (NCCP), ParsTechRokh Inc. Mashhad, Iran

**Keywords:** Human Y-Chromosome

## Abstract

Chromosome-centric human proteome project (C-HPP) is a recent initiative to rationalize and analyze gene-protein and protein-protein interactions in normal and disease conditions. This initiative is aimed to generate the proteomic atlas explaining the molecular architecture of the human body and was initiated in response to the hurdles identified during analyzing the human genome project (HGP). A need for the experimental observation of translated proteins was felt to analyze precisely what is going on in the cell. 25 countries around the world are participating in the C-HPP. This symposium report will introduce the Y-chromosome HPP which is undergoing in Iran by eminent molecular biologists of Royan Institute, Tehran and its collaborates.

## Introduction

**Fig 1:**
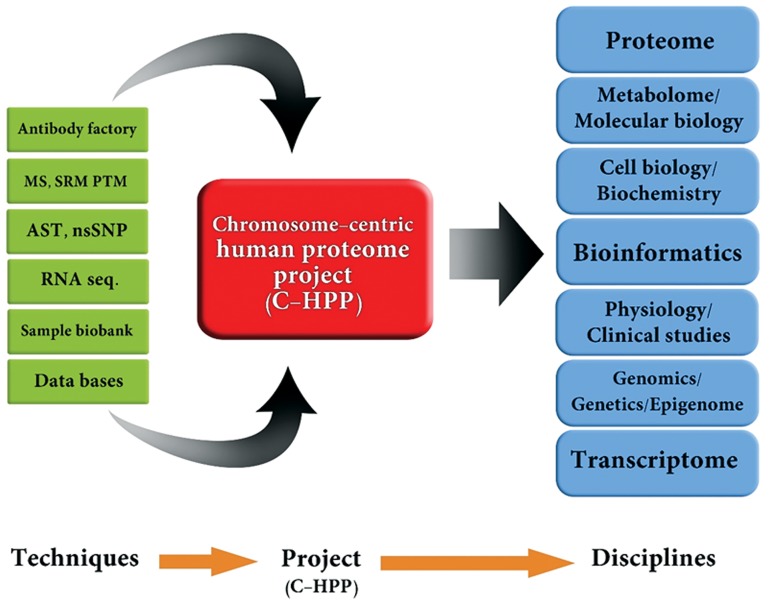
Components of the chromosome-centric human proteome project (C-HPP) research module (5). MS; Mass spectrometry, SRM PTM; Selected reaction monitoring post translational modification, AST; Alternative splicing transcript and nsSNP; Nonsynonymoussingle-base nucleotide polymorphism.

The human proteome project (HPP) is a systematic global effort to analyze the molecular behavior of translated proteins, their distribution and localization, and interactions and functions in the human body. In 2011, complete directions of the project such as its future exploration and its current status were explained (1). After finalizing the directions and targets, scientists explained that this project will be the effective integration of proteomics data into a genomic framework that will help us understand complex biological systems and to predict protein-based solutions to chronic diseases as it will catalog the proteins encoded by the genome as shown in figure 1 (2). Twenty-three pairs of human chromosomes along with the mitochondrial chromosome are divided among 25 countries around the world. X- and Y-chromosomes are independently assigned to Japan and Iran respectively (Table 1). Journal of Proteome Research has assigned a specific annual issue C-HPP (12:1, 2013 and 13:1, 2014) to uncover the key developments in C-HPP and the current status of overall project has also been discussed in both issues (3, 4).

In line with research activities of Y-C-HPP, the first Y Chromosome Proteome Project Symposium was held in Royan Institute. More than 200 students and researchers attended this symposium. In this report, the scientific program of the symposium has been categorized into 4 sections. First section was regarding the introduction of Royan Institute and the role of the Islamic Republic of Iran in Y-HPP. In section two, the invited speakers discussed the need for C-HPP and presented a 2014-update on XY-HPP. The third section was the presentations of students and researchers on the work they are under taking and highlighting their current results and future directions. The last section was the panel discussion among the speakers and participants.

## Molecular biological research in Iran

It is for the first time that an Islamic country is participating in a global molecular biological project and Iran is thus proving itself to be a pioneer in the Islamic world (5). Iranian proteomic society, a member of Human Proteome Organization, bid for taking the Y chromosome and to be a contributing participant of C-HPP.

**Table 1:** Chromosome-centric national affiliations in chromosome-centric human proteome project (C-HPP)


Chromosome	1	2	3	4	5	6	7	8	

	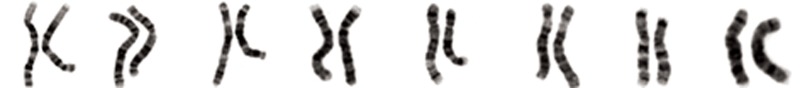								
**National affiliations**	China	Switzer-land	Japan	Taiwan	Nether-lands	Canada	Austral-ia, NewZealand	China	
**Chromosome**	9	10	11	12	13	14	15	16	
	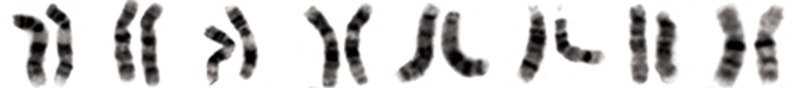								
**National affiliations**	Seoul,Korea	USA	Korea	India,Singa-pore,Taiwan,Thailand	Korea	France	Brazil	Spain	
**Chromosome**	17	18	19	20	21	22	X	Y	Mito-chondrial
	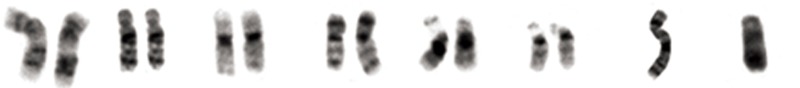								
**National affiliations**	USA	Russia	Sweden	China	Canada	USA	Japan	Iran	Italy


## Section 1 and 2

1^st^ symposium on Y-CHPP was chaired by Dr. Gh. Hosseini Salekdeh who is also the president of Iranian Proteomic Society and also the head of Y-chromosome HPP. After a brief introduction on Royan Institute by Dr. H. Gourabi (Royan Institute president), eminent molecular biologists presented theories and perspectives on X- and Y-HPP. Dr. M. Sedighi Gilani presented the "[g] eneralities about clinical topics in the world of infertility" as the first talk of the symposium and related Y-HPP with a solution for infertility in the world. The talk by Dr. M. Nasr-Esfahani was on the assessment of infertile men as potential candidates for artificial oocyte activation. Dr. N. Ansari Pour then provided an evolutionary perspective on Y-chromosomal variation and its relation to male infertility. Finally, Dr. T. Yamamoto, the head of X-HPP and Dr. Gh. Hosseini Salekdeh (Head of Y-HPP) discussed the 2014-updates of X- and Y-HPP, respectively. Protein- protein interaction and bioinformatics-based work and their potential to rationalize the experimental results of Y-HPP were presented by Dr. S. Hosseinkhani and Dr. M. Sadeghi respectively.

## Sections 3 and 4

After the X- and Y-HPP were introduced, young researchers working on this project presented the on-going work on Y-HPP. Different topics regarding Y-chromosomal associated diseases and the proteins involved were presented including role of lysine-specific demethylase 5D (*Y-chromosome* gene) in prostate cancer, role of AZFb region genes in azoospermia, androgenic treatments to NT2 cells, and role of Y-chromosome male specific region genes and their X-linked homologs in dimorphic development of brain. Inter-relation of C-HPP and human pluripotent stem cells was one of the most interesting topics for many participants. An extensive participation was observed in the panel discussion regarding the outcomes of C-HPP. The next plausible global project that may be essential in the future was also discussed if CHPP does not solve human biological problems and that a gap between the human genome project and human proteome projectstays relatively unchanged.

## Conclusion

The quest to identify solutions to human diseases and biological problems is a century-old project. Anatomical and physiological knowledge of the human species gave us clues to what goes on in the human body but still we were far away from real causes of diseases and as we were far away from the real causes of diseases, we were far away from their possible solutions. In the 90s, based on the hypothesis that DNA is the code of life, HGP was initiated. After the completion of HGP, the question of "what is going on in the cell" remained unsolved. In 2011, the C-HPP was started to find out how cells perform their functons. In the discussion section of this symposium, I personally, discussed with Dr. Yamamoto about possible questions that could remain unsolved e.g. the proteomic analysis would answer the evolutionary modifications of proteins or domain arrangement of proteins. A possible solution to the gap between HGP and CHPP was also discussed and it was argues that in future, we may need the human transcriptome project (HTP) to fill this gap. Unravelling the genome to transcriptome to proteome pathway may be the answer to all questions that have remained unanswered.
